# The skåne emergency medicine (SEM) cohort

**DOI:** 10.1186/s13049-024-01206-0

**Published:** 2024-04-26

**Authors:** Ulf Ekelund, Bodil Ohlsson, Olle Melander, Jonas Björk, Mattias Ohlsson, Jakob Lundager Forberg, Pontus Olsson de Capretz, Axel Nyström, Anders Björkelund

**Affiliations:** 1 Emergency medicine, Department of Clinical Sciences Lund, Lund University, Department of Emergency Medicine, Skåne University Hospital, Lund, Sweden; 2 Department of Clinial Sciences Malmö, Lund University, Department of Internal Medicine, Skåne University Hospital, Malmö, Sweden; 3 Occupational and Environmental Medicine, Department of Laboratory Medicine, Lund University, Lund, Sweden; 4 Forum South, Clinical Studies Sweden, Skåne University Hospital, Lund, Sweden; 5 Emergency medicine, Department of Clinical Sciences Lund, Lund University, Department of Emergency Medicine, Helsingborg Hospital, Helsingborg, Sweden; 6 Centre for Environmental and Climate Science, Lund University, Lund, Sweden; 7 Center for Applied Intelligent Systems Research (CAISR), Halmstad University, Halmstad, Sweden

**Keywords:** Emergency medicine, Emergency department, Database, Decision-making, Artificial intelligence, Machine learning

## Abstract

**Background:**

In the European Union alone, more than 100 million people present to the emergency department (ED) each year, and this has increased steadily year-on-year by 2–3%. Better patient management decisions have the potential to reduce ED crowding, the number of diagnostic tests, the use of inpatient beds, and healthcare costs.

**Methods:**

We have established the Skåne Emergency Medicine (SEM) cohort for developing clinical decision support systems (CDSS) based on artificial intelligence or machine learning as well as traditional statistical methods. The SEM cohort consists of 325 539 unselected unique patients with 630 275 visits from January 1st, 2017 to December 31st, 2018 at eight EDs in the region Skåne in southern Sweden. Data on sociodemographics, previous diseases and current medication are available for each ED patient visit, as well as their chief complaint, test results, disposition and the outcome in the form of subsequent diagnoses, treatments, healthcare costs and mortality within a follow-up period of at least 30 days, and up to 3 years.

**Discussion:**

The SEM cohort provides a platform for CDSS research, and we welcome collaboration. In addition, SEM’s large amount of real-world patient data with almost complete short-term follow-up will allow research in epidemiology, patient management, diagnostics, prognostics, ED crowding, resource allocation, and social medicine.

**Supplementary Information:**

The online version contains supplementary material available at 10.1186/s13049-024-01206-0.

## Background

All over the world, emergency departments (ED) are struggling with an increasing inflow of patients, and especially elderly patients with complex pathology that is difficult to assess due to simultaneous chronic diseases, risk factors and/or polypharmacy [1, 2]. ED clinicians need to make fast and accurate risk estimates, and optimal management from the start is crucial for good patient outcomes. At the same time, the amount of available clinical information in electronic medical records is also increasing, as is the total body of medical knowledge. Often the ED physician can no longer grasp and process all available information, making it impossible for an individual clinician to provide the theoretically best possible care.

Artificial intelligence (AI) and machine learning (ML) are now developing fast, and most industries will likely be fundamentally changed by AI in the coming years [3]. In medicine, AI and ML provide new possibilities when applied to extensive electronic health records and registers [4]. The most impressive advances have occurred in radiology and pathology, where ML accuracy of image classifications now exceeds that of humans [5]. In emergency medicine, AI/ML-driven decision support tools have the potential to improve diagnostic accuracy [5], alleviate ED crowding [6, 7], and decrease the use of inpatient beds and healthcare costs [8]. The Swedish Board of Health and Welfare has therefore emphasized the great potential of AI/ML in emergency medicine [9]. So far however, there have been few AI/ML studies in the ED setting, and practically no implementation in routine ED care. The creation of ML-based decision support for ED use requires large amounts of high-quality clinical data, preferably from representative unselected ED patients in routine care.

In the present paper we describe the rationale for, and construction of, the Skåne Emergency Medicine (SEM) cohort and outline possible studies. The SEM cohort is a recently established data platform for developing clinical decision support systems (CDSS) based on traditional statistical methods or AI/ML, to be used in ED triage or later in the management of specific patient conditions. Specific aims include the prediction of diagnoses, critical interventions (e.g. defibrillation of cardiac arrest, thrombolysis in stroke) or inpatient care within 30 days of the ED visit, and mortality up to 1 year after the ED visit. We describe in this paper the process of building the SEM dataset with careful consideration of ethics, data protection, and bias. With the SEM cohort, we hope to create CDSS that can be tested in randomized trials in routine emergency care.

## Methods/design

The formation of the SEM cohort was an initiative within the Artificially Intelligent use of Registers at Lund University (AIR Lund) research environment [10], which is a multidisciplinary collaboration between Lund University (Emergency medicine, Internal medicine, Epidemiology and biostatistics, Computational biology, Technology and society/ethics, and Law), Halmstad University (Information technology), and the Swedish health care regions Skåne and Halland.

### Setting

Skåne is Sweden’s southernmost region and has some 1.4 million inhabitants. Healthcare is publicly financed with a small copayment at every visit. Patients in region Skåne almost always go to the nearest ED, and in general do not seek care outside the region. The SEM cohort includes data from patients presenting at eight general EDs in Skåne from January 1st, 2017 to December 31st, 2018. The characteristics of these EDs are described in Table 1. Five EDs are open 24/7/365 (Skåne university hospital at Lund and Malmö, Helsingborg general hospital, Kristianstad central hospital and Ystad hospital) and three EDs are open during office hours (Landskrona, Trelleborg and Hässleholm hospitals). There are very few patients with psychiatric disorders, problems related to obstetrics/ gynecology, ophthalmology, and pediatric patients without orthopedic problems at these EDs, since there are specialized EDs for these patients in the region. Table 1 describes that the yearly ED census ranges between 80000 (Malmö) and 5000 (Landskrona) patient cases, and that admission rates to in-hospital care range between 20% (Helsingborg) and 32% (Hässleholm or Landskrona). All EDs use the rapid emergency triage and treatment system (RETTS [11]) that includes five priority levels: Highest priority 1 (Red); Priority 2 (Orange); Priority 3 (Yellow); Lowest priority 4 (Green); and Priority primary care (Blue). The RETTS set of chief complaints are thus common for all EDs in the SEM cohort. All EDs have similar access to patient testing, and clinical guidelines are generally the same in the entire region.

**Table 1:** Characteristics of the EDs included in the SEM cohort, after Welch et al.[21] *trauma level according the American College of Surgeons [22]. EM, emergency medicine; ENT, Ear nose and throat; Ob/Gyn, Obstetrics/Gynecology

Hospital	Number of patient visits in SEMD	Admission rate, %	Trauma level*	Specialties present (patient spectrum received)	Open 24/7/365	Transplant Service in hospital	Acuity	EM specialist training program
**Malmö**	157 825	25	2	Internal Medicine, Neurology, Surgery, Urology, Orthopedics & Trauma, Infectious diseases, Pediatrics, ENT	*Yes*	*Yes*	*High*	*Yes*
**Helsingborg**	141 621	20	2	Internal Medicine, Neurology, Surgery, Urology, Orthopedics & Trauma, Infectious diseases, Pediatrics, ENT, Urgent primary care, Ophtalmology	*Yes*	*No*	*High*	*Yes*
**Lund**	128 851	26	1	Internal Medicine, Neurology, Surgery, Urology, Orthopedics & Trauma, Infectious diseases, ENT	*Yes*	*Yes*	*High*	*Yes*
**Kristianstad**	95 690	24	2	Internal Medicine, Neurology, Surgery, Urology, Orthopedics & Trauma, Infectious diseases, Pediatrics, Ob/Gyn, ENT	*Yes*	*No*	*High*	*Yes*
**Ystad**	52 078	30	3	Internal Medicine, Neurology, Surgery, Urology, Orthopedics & Trauma, Infectious diseases, Pediatrics, ENT, Ob/Gyn, Ophtalmology	*Yes*	*No*	*High*	*Yes*
**Trelleborg**	24 704	25	3	Internal Medicine, Neurology, Surgery, Urology, Orthopedics & Trauma, Infectious diseases	*No*	*No*	*High*	*No*
**Hässleholm**	18 905	32	No trauma patients	Internal Medicine, Neurology	*No*	*No*	*Low*	*No*
**Landskrona**	10 210	32	No trauma patients	Internal Medicine	*No*	*No*	*Low*	*No*

During and after the data collection period, the patients were informed of the purpose and structure of the SEM cohort in writing via public advertising on a website, and that they could decline participation at any time, for any reason, by contacting a research nurse or the first author at Lund. The creation of the SEM cohort and its use for AI/ML research and cross-sectional analyses has been approved by the Swedish Ethical Review Authority (Dnr 2019–05783), and by Region Skåne (302 − 19). There is no approval for commercial use of the data.

### Data collection

During the study period, all patients at the eight EDs were included in the SEM cohort by default via identification in the common ED patient log system (Patientliggaren™, Tietoevry [12]), and data from the other registers (below) were then linked by each patient’s unique Swedish identification (ID) number, which is universally used in Swedish healthcare and all government registers. After collection and linkage, all data were pseudonymized with patient study ID numbers and kept on secure servers behind firewalls at Lund University where access is logged. The key between personal and study IDs is kept separately on a Region Skåne server with standard healthcare data security.

The data sources include healthcare databases and registers with complete national or regional coverage, which should ensure close to complete data on all patient visits. As much as possible, we used well described high-quality data sources (see e.g. references [13–16]) to collect the SEM data in order to decrease bias and data errors. The number of missing data varies across the sources but is generally very low. Data variables were chosen based on importance in the emergency care process as well as availability in the source registers. The collected data were the same as used in clinical care, and there was no major change in data labelling during 2017–2018. The SEM cohort was not designed with a specific CDSS or study in mind, but the size of the cohort (below) and the number of variables and data included was chosen to ensure sufficient statistical power for most CDSS research projects.

Data from the source registers were kept in their exported form with no deletion or curation, and software scripts are used to extract data to form tailor-made new datasets for each specific research project. Data curation or deletion will generally take place in each CDSS project, and only as needed in the original SEM cohort data.

As shown in Table 2, the available data for each patient visit include the patient’s baseline data, data on the ED visit, and the outcome within 30 days up to three years after the ED visit: diagnoses, ED returns, hospital admissions, death, and healthcare costs. In total, the SEM data include several hundred variables for each patient, and many more that can be calculated from the original variables, such as ED crowding or boarding data, return visits, and mortality at different times after ED arrival. Detailed variable lists are available on reasonable request.

**Table 2:** Available data for each patient visit in the SEM cohort

Type of data	Description and examples of variables/ variable groups	Source
**Baseline data**		
Basic and sociodemographic patient characteristics	Age, sex, country of birth, education, marital status, place of residence, income, welfare benefits etc.	[1]
Previous disease	Diagnoses (ICD10 codes) in the region during 5 years before ED visit	[8]
Previous tests	Diagnostic/ prognostic tests in the region during 5 years before the ED visit	[8], [9], [10], [11], [12]
Current medication	Prescriptions redeemed in Sweden during one year before ED visit	[2]
***Data on the ED visit***		
Chief complaint	As defined by the RETTS system [11]	[7]
ED throughput times	ED arrival time and date, time to ED nurse, ED physician, ED length of stay, ED discharge time and date.	[6], [7]
Findings at the physical examination, vital signs	Free text, available by manual review of the patient records	[8]
Medications	Given at the ED (free text) and after the ED but within 24 h after ED presentation (digitized)	[8]
Diagnostic/prognostics tests	Performed within 24 h after ED presentation, including ECG, imaging, functional tests etc., with free text results	[8], [9], [10], [11], [12]
Disposition	Left without being seen, admission to in-hospital care.	[7]
Preliminary diagnosis/ assessment at the ED	ICD10 codes and free text	[8]
***Outcome data***		
In-hospital admission ward	Name (and type) of in-hospital ward	[8]
Hospital length of stay		[8]
Intensive care	Intensive care admissions/interventions within 30 days after ED presentation	[8]
Diagnoses	To the end of 2019	[8]
Critical interventions/ treatments	Cardiac defibrillation, thrombolysis, percutaneous coronary intervention etc. to end of 2019	[8]
ED returns	Re-presentations at ED up to end of 2019	[7]
Hospital admissions	Admissions to in-hospital care and length of stay to end of 2019	[3], [8]
Date of death	To end of 2019	[4], [6]
Cause of death	To end of 2019	[5]
Healthcare costs	Direct healthcare costs in the region within 30 days after the ED visit.	[13]

**SEM data sources**

*Registers and databases with national (Sweden) coverage*

1. Statistics Sweden; [13] The LISA database, the Geography database

2. National Board of Health and Welfare; National Prescribed Drug Register [15, 23]

3. National Board of Health and Welfare; National Patient Register; [14, 24]

4. The Swedish population register [13]

5. National Board of Health and Welfare; National Cause of Death Register [16, 25]

6. Swedish emergency care register; SVAR [26–28]

*Region Skåne registers and databases with complete regional coverage*

7. The ED patient log system (Patientliggaren™, Tietoevry [12])

8. The electronic healthcare records (Melior™, Siemens [29]) and/or the Patient administrative system (PASIS)

9. The ECG-database (MUSE™, GE Healthcare [30])

10. The imaging and functional testing database (SECTRA [31])

11. The clinical chemistry database (LIMS RS)

12. The microbiology database

13. The healthcare economics system

The SEM cohort is thus mainly based on register data and does not include free text information such as the patient’s detailed symptom history, findings at the physical examination, reasons for decisions and preliminary assessments. Also missing are the initial ED vital signs (blood oxygen saturation, respiratory rate, pulse rate, blood pressure, consciousness level and body temperature) and pharmacological treatment in the ED, since these data are primarily recorded on paper in the region. However, all this missing information can be obtained as needed by manual review of the individual patient records. As for diagnostic tests, ECG data are available as the raw signal, amplitude/interval measurements as well as the machine interpretation, and imaging and functional test data are available as the free text results. The images are not part of the SEM cohort data but can be obtained in specific projects.

### Basic cohort characteristics

The SEM cohort is briefly described in Table 3 and includes 325 539 unique patients with 630 275 ED visits during 2017 and 2018. Fewer than five patients declined participation which makes the cohort almost 100% complete. The mean age was 55 years, 49% were male and 23.5% of all patients arrived by ambulance. The most common triage category was 3, Yellow, and 15.0% of the patients had no registered triage category mostly due to immediate referral from the ED to external primary care or self-care. 11% of the patients had previous diagnoses of diabetes, 10% of cancer, 8% of pulmonary disease, and 1.7% suffered from dementia.

**Table 3:** Baseline patient characteristics and management in the SEM cohort. Std, standard deviation. *Among the unique patients

Patient visits, n	630275
Unique patients, n	325539
Male, n, %	308990, 49.0%
Age, mean (std)	55.2 (21.8)
Arrival by ambulance, n, %	147976, 23.5%
**Triage category**	
Highest priority; 1. Red	5.8%
Priority 2. Orange	20.4%
Priority 3. Yellow	46.4%
Lowest priority; 4. Green	6.3%
Primary care, Blue	6.1%
Missing	15.0%
Time to doctor in min, median (iqr)	70.0 (32.0-137.0)
ED length of stay in min, median (iqr)	206.0 (99.0-346.0)
ECG registered	29.4%
Imaging performed within 24 h	41.4%
Admitted to in-hospital care	24.4%
ED revisit in 7d	13.8%
**Previous diagnoses (ICD10 codes) at ED presentation**	Prevalence*
Diabetes (E109, E119, E139, E149, E101, E111, E131, E141, E105, E115, E135, E145)	11.0%
Cancer (C0-3, C40-49, C5-6, C70-76, C80-85, C883, C887, C889-901, C91-93, C940-943, C9451, C947, C95-96)	10.3%
Pulmonary disease (J40-47, J60-67)	8.2%
Cerebrovascular incident (I60-63, I65-66, G450-452, G454, G458-459, G46, I64, I670-672, I674 I675-679, I681-682 I688, I69)	7.3%
Congestive heart failure (I50)	7.0%
Renal disease (N01, N03, N052-056, N072-074, N18-19, N25)	4.0%
Peripheral vascular disease (I71, I790, I739, R02, Z958, Z959)	2.6%
Dementia (F00-02, F051)	1.7%

Table 4 shows that the most common chief complaint in SEM was abdominal pain, followed by chest pain, dyspnea, hand injury and unspecific disorder. (The term “unspecific disorder” is used when the triage nurse is unable to classify the patient’s problem using the more specific terms in the system.) Some 9% of all visits had no registered chief complaint, again mostly because of immediate referral to primary or self-care. The median time to doctor was 70 min and the median length of stay was 206 min. In 24% percent of all ED visits the patient was admitted to in-hospital care.

**Table 4:** Twenty most common chief complaints in the SEM cohort, according to the RETTS system [11]. The term “Unspecific disorder” is used when the triage nurse is unable to classify the patient’s problem using the more specific terms in the system

	Number of patient visits	% of all patients
Abdominal pain	67,171	10.7%
Missing	57,462	9.1%
Chest pain	51,351	8.1%
Dyspnea	38,154	6.1%
Injury, hand	24,507	3.9%
Unspecific disorder	23,302	3.7%
Extremity pain	19,814	3.1%
Injury, head	18,932	3.0%
Injury foot	16,255	2.6%
Dizziness	15,767	2.5%
Infection	15,451	2.5%
Arrhythmia	14,350	2.3%
Neurological deficit	13,498	2.1%
Fever	12,945	2.1%
Headache	12,288	1.9%
Back pain	11,622	1.8%
Extremity symptom	10,905	1.7%
Renal colic	9939	1.6%
Injury, knee	9566	1.5%

As can be seen in Table 5, the most common discharge diagnoses were bacterial pneumonia, cerebrovascular incident, and acute myocardial infarction. The mortality at the ED was 0.2%, it was 0.9% within 7 days, and 2.2% within 30 days.

**Table 5:** Selected discharge diagnoses from the ED or from in-hospital care directly following the ED visit, in the SEM cohort

Discharge diagnoses	ICD-10 codes	n, % of all patients
Bacterial Pneumonia	J13-15, J18	17,047, 2.7%
Cerebrovascular incident	I60-63, I65-66, G450-452, G454, G458-459, G46, I64, I670-672, I674 I675-679, I681-682 I688, I69	15,400, 2.4%
Acute myocardial infarction	I21, I22	14,258, 2.3%
Sepsis	A021, A207, A227, A392, A327, A394, A40, A41, R572, R65	6589, 1.0%
Intoxication	F100, F110, F120, F130, F140, F150, F160, F170, F180, F190	4851, 0.8%
Hip Fracture	S720-722	4267, 0.7%
Ileus	K56	3400, 0.5%
Pulmonary Embolism	I26	3113, 0.5%
Cholecystitis	K800-801, K804, K81	2955, 0.5%
Acute Appendicitis	K35, K36, K37	2623, 0.4%
Pancreatitis	K85	1759, 0.3%
Aortic Dissection	I710	204, 0.03%
**Death**		
Death at the ED		1004, 0.2%
Death within 7 days of ED arrival		5969, 0.9%
30 days		13,755, 2.2%
365 days		50,908, 8.1%

## Discussion

In addition to CDSS development, SEM’s large amount of real-world ED patient data with almost complete follow-up will allow research in many fields of emergency medicine: Epidemiology, patient management, diagnostics, prognostics, ED crowding, resource allocation, and social medicine. Some of these studies may need supplementary ethics approval. The SEM cohort is currently being used to analyze cases of missed acute aortic syndrome, for prediction of venous thromboembolism, mapping of characteristics and outcomes in patients with dizziness or with head trauma, and for the evaluation of emergency care for adult patients with congenital heart disease.

Studies of the epidemiology of ED patients may be beneficial for public health surveillance, resource planning, evaluating healthcare delivery and for facilitating research, e.g. sample size calculations for prospective studies. Epidemiological information supports clinical evidence-based decision-making and enables the ED to organize according to the needs of the population. The SEM cohort includes almost all patients presenting at eight EDs in southern Sweden during two years, and it should therefore be possible to obtain reasonably accurate and generalizable data on chief complaints and underlying disease states in the entire population as well as in subgroups based on age, sex, comorbidities or sociodemographics. Also, diurnal, weekly, and seasonal variations may be described.

ED patient management and its impact on outcomes may be studied in the SEM cohort by analyzing e.g. waiting times, length of ED stay, admissions to intensive care, as well as patients who left without being seen by a physician or who returned to the ED. These analyses may also be made in the absence or presence of ED crowding. As mentioned, pharmaceutical treatment at the ED is not immediately available but can be extracted for all patients from the digitized (scanned) ED patient paper records.

The SEM cohort allows analysis of the accuracy of diagnostic and functional testing by comparing pre-test probability with short or medium-term outcomes such as diagnoses or death.

The utilization and costs of diagnostic testing, hospital admission and care at specific wards in each patient up to 30 days in the cohort can be used to analyze resource use in all patients and in specific subgroups. Also, the SEM cohort may be used to evaluate ED care and acute healthcare consumption in different socioeconomic and demographic groups, as well as inequalities and possible discrimination.

## Strengths and limitations

SEM includes real-world clinical data from consecutive patients presenting to eight different EDs during two years. The large number of patient visits, variables, and clinical events should be sufficient for most analyses of interest. Data were collected in regular care and there are several general advantages with using routine care data when building CDSS. Firstly, it provides access to large amounts of data from a diverse and unselected patient population, which is crucial for developing CDSS that work across different patient demographics. Secondly, routine care data may be immediately available, reducing the cost and time required to collect data. Finally, routine care data collection will often allow simple tracking of patient outcomes and evaluation of the effectiveness of the CDSS, especially in a country with comprehensive healthcare databases like Sweden. In the future, it may be possible to use native, uncurated electronic health records directly for medical research [17]. Another strength of the multimodal SEM cohort is its potential utility in developing CDSS that provide relative risks of multiple diagnoses, in contrast to algorithms based on a single type of input and output (e.g. radiology algorithms detecting cancer), and current clinical decision support tools which often serve merely as rule-out tests, e.g. the PERC rule for pulmonary embolism.

SEM includes data from ED patient visits in one Swedish region, and the data may therefore not be generalizable to other populations or healthcare settings. There are few patients in the SEM cohort with problems related to psychiatry, obstetrics/gynecology, and ophthalmology, as well as few pediatric patients without orthopedic problems. Some clinical variables are missing or less readily available in SEM, e.g. free text imaging results that require manual review, and this will of course prevent or complicate the creation of some types of CDSS, as well as some data disaggregation. Missing data in SEM are rare, but there may of course be errors in the data, which can lead to biased or inaccurate CDSS. Since SEM data were registered as part of regular care, bias may also arise from different patient evaluation and management based on previous clinical findings (verification bias) or based on patients’ ethnic or socioeconomic background. Also, historical bias will exist in any clinical database, i.e. when the data no longer accurately reflect a new healthcare reality.

Several variables in the SEM database were originally manually entered or determined subjectively, such as time stamps in the ED and discharge diagnoses and may therefore contain errors or bias. Diagnoses might also have been registered several times for the same care episode. Bias or errors in the training data will cause a high risk of bias in the final CDSS, but the size and impact of the problem will vary in different CDSS. The optimal approach to the potential problem with bias is therefore best determined in each use case and CDSS. Before clinical implementation, any CDSS based on SEM data should be carefully reviewed and prospectively tested in a clinical trial in the specific healthcare setting.

On the other hand, it should be noted that if a CDSS is intended to operate in real time with standard register data as input, it is preferable that the underlying ML model is developed using this type of data rather than curated data that do not reflect the “dirty” truth of day-to-day operations. With sufficiently large training data, current ML algorithms can cope with a fair amount of noise and navigate between varying levels of noise in different types of input data.

In addition to algorithm quality, several barriers to successful implementation and use of AI/ML-based CDSS must be considered: IT problems, low model transparency (black box algorithms), proprietary code, lack of trust and knowledge among physicians and decision-makers, legal framework (oversight, malpractice issues) and ethical issues, integrity risks and financial challenges [18–20]. However, the size and implications of these barriers will vary in different use cases.

In conclusion, the SEM cohort provides a platform for collaborative CDSS research. SEM’s large amount of real-world patient data with almost complete follow-up will also allow research in epidemiology, patient management, diagnostics, prognostics, ED crowding, resource allocation, and social medicine.

### SEM cohort access

So far, collaborations have been established with other research groups at Lund and Halmstad Universities in Sweden. We welcome initiatives on international collaborative projects using the SEM cohort. Anonymized parts of the SEM database will be available for sharing on reasonable request, as will detailed variable lists. Please contact the corresponding author via email (ulf.ekelund@med.lu.se).

### Electronic supplementary material

Below is the link to the electronic supplementary material.

Supplementary Material 1

Supplementary Material 2

## Data Availability

Anonymized parts of the SEM database will be available for sharing on reasonable request. Please send an email to ulf.ekelund@med.lu.se.

## Abbreviations

AI: artificial intelligence
CDSS: clinical decision support system
ED: emergency department
ML: machine learning
RETTS: rapid emergency triage and treatment system
SEM cohort: Skåne emergency medicine cohort
