# Supplementary material for: The skåne emergency medicine (SEM) cohort
Source: Scand J Trauma Resusc Emerg Med. 2024 Apr 26;32:37. doi: 10.1186/s13049-024-01206-0 (PMC11046860; doi:10.1186/s13049-024-01206-0)
Supplement: Supplementary file 2 — Supplementary Material 2 [file 13049_2024_1206_MOESM2_ESM.docx]

**The Skåne Emergency Medicine (SEM) cohort**

Ulf Ekelund MD, PhD ulf.ekelund@med.lu.se

Emergency medicine, Department of Clinical Sciences Lund, Lund University, Skåne University Hospital, Lund, Sweden

Bodil Ohlsson MD, PhD bodil.ohlsson@med.lu.se

Internal medicine, Department of Clinical Sciences Malmö, Lund University, Skåne University Hospital Malmö, Sweden

Olle Melander MD, PhD olle.melander@med.lu.se

Internal medicine, Department of Clinical Sciences Malmö, Lund University, Skåne University Hospital Malmö, Sweden

Jonas Björk PhD jonas.bjork@med.lu.se

Occupational and Environmental Medicine, Department of Laboratory Medicine, Lund University, Lund, Sweden. Clinical Studies Sweden, Forum South, Skåne University Hospital, Lund, Sweden

Mattias Ohlsson PhD mattias.ohlsson@cec.lu.se

Centre for Environmental and Climate Science, Lund University, Lund, Sweden. Center for Applied Intelligent Systems Research (CAISR), Halmstad University, Sweden

Jakob Lundager Forberg MD, PhD Jakob.Lundager-Forberg@skane.se

Emergency medicine, Department of Clinical Sciences Lund, Lund University, Helsingborg Hospital, Helsingborg, Sweden

Pontus Olsson de Capretz MD pontus.olsson86@gmail.com

Emergency medicine, Department of Clinical Sciences Lund, Lund University, Skåne University Hospital, Lund, Sweden

Axel Nyström MSc axel.nystrom@med.lu.se

Centre for Environmental and Climate Science, Lund University, Lund, Sweden

Anders Björkelund PhD anders.bjorkelund@cec.lu.se

Centre for Environmental and Climate Science, Lund University, Lund, Sweden

**Corresponding author:** Ulf Ekelund (ulf.ekelund@med.lu.se)

**Key words**: Emergency medicine, emergency department, database, decision-making, artificial intelligence, machine learning.

**Word count**: 2727 (main text, not including abstract, references, tables, legends)

**Abstract**

***Background.*** In the European Union alone, more than 100 million people present to the emergency department (ED) each year, and this has increased steadily year-on-year by 2-3%. Better patient management decisions have the potential to reduce ED crowding, the number of diagnostic tests, the use of inpatient beds, and healthcare costs.

***Methods.*** We have established the Skåne Emergency Medicine (SEM) cohort for developing clinical decision support systems (CDSS) based on artificial intelligence or machine learning as well as traditional statistical methods. The SEM cohort consists of 325 539 unselected unique patients with 630 275 visits from January 1^st^, 2017 to December 31^st^, 2018 at eight EDs in the region Skåne in southern Sweden. Data on sociodemographics, previous diseases and current medication are available for each ED patient visit, as well as their chief complaint, test results, disposition and the outcome in the form of subsequent diagnoses, treatments, healthcare costs and mortality within a follow-up period of at least 30 days, and up to 3 years.

***Discussion.*** The SEM cohort provides a platform for CDSS research, and we welcome collaboration. In addition, SEM’s large amount of real-world patient data with almost complete short-term follow-up will allow research in epidemiology, patient management, diagnostics, prognostics, ED crowding, resource allocation, and social medicine.

**Keywords**

Emergency department, decision making, decision support, machine learning, artificial intelligence, epidemiology.

**List of abbreviations**

AI, artificial intelligence

CDSS, clinical decision support system

ED, emergency department

ML, machine learning

RETTS, rapid emergency triage and treatment system

SEM cohort, Skåne emergency medicine cohort

#### **Background**

All over the world, emergency departments (ED) are struggling with an increasing inflow of patients, and especially elderly patients with complex pathology that is difficult to assess due to simultaneous chronic diseases, risk factors and/or polypharmacy.^1,2^ ED clinicians need to make fast and accurate risk estimates, and optimal management from the start is crucial for good patient outcomes. At the same time, the amount of available clinical information in electronic medical records is also increasing, as is the total body of medical knowledge. Often the ED physician can no longer grasp and process all available information, making it impossible for an individual clinician to provide the theoretically best possible care.

Artificial intelligence (AI) and machine learning (ML) are now developing fast, and most industries will likely be fundamentally changed by AI in the coming years.^3^ In medicine, AI and ML provide new possibilities when applied to extensive electronic health records and registers.^4^ The most impressive advances have occurred in radiology and pathology, where ML accuracy of image classifications now exceeds that of humans.^5^ In emergency medicine, AI/ML-driven decision support tools have the potential to improve diagnostic accuracy,^5^ alleviate ED crowding,^6,7^ and decrease the use of inpatient beds and healthcare costs.^8^ The Swedish Board of Health and Welfare has therefore emphasized the great potential of AI/ML in emergency medicine.^9^ So far however, there have been few AI/ML studies in the ED setting, and practically no implementation in routine ED care. The creation of ML-based decision support for ED use requires large amounts of high-quality clinical data, preferably from representative unselected ED patients in routine care.

In the present paper we describe the rationale for, and construction of, the Skåne Emergency Medicine (SEM) cohort and outline possible studies. The SEM cohort is a recently established data platform for developing clinical decision support systems (CDSS) based on traditional statistical methods or AI/ML, to be used in ED triage or later in the management of specific patient conditions. Specific aims include the prediction of diagnoses, critical interventions (e.g. defibrillation of cardiac arrest, thrombolysis in stroke) or inpatient care within 30 days of the ED visit, and mortality up to 1 year after the ED visit. We describe in this paper the process of building the SEM dataset with careful consideration of ethics, data protection, and bias. With the SEM cohort, we hope to create CDSS that can be tested in randomized trials in routine emergency care.

**Methods/design**

The formation of the SEM cohort was an initiative within the Artificially Intelligent use of Registers at Lund University (AIR Lund) research environment,^10^ which is a multidisciplinary collaboration between Lund University (Emergency medicine, Internal medicine, Epidemiology and biostatistics, Computational biology, Technology and society/ethics, and Law), Halmstad University (Information technology), and the Swedish health care regions Skåne and Halland.

*Setting*

Skåne is Sweden’s southernmost region and has some 1.4 million inhabitants. Healthcare is publicly financed with a small copayment at every visit. Patients in region Skåne almost always go to the nearest ED, and in general do not seek care outside the region. The SEM cohort includes data from patients presenting at eight general EDs in Skåne from January 1^st^, 2017 to December 31^st^, 2018. The characteristics of these EDs are described in Table 1. Five EDs are open 24/7/365 (Skåne university hospital at Lund and Malmö, Helsingborg general hospital, Kristianstad central hospital and Ystad hospital) and three EDs are open during office hours (Landskrona, Trelleborg and Hässleholm hospitals). There are very few patients with psychiatric disorders, problems related to obstetrics/ gynecology, ophthalmology, and pediatric patients without orthopedic problems at these EDs, since there are specialized EDs for these patients in the region. Table 1 describes that the yearly ED census ranges between 80000 (Malmö) and 5000 (Landskrona) patient cases, and that admission rates to in-hospital care range between 20% (Helsingborg) and 32% (Hässleholm or Landskrona). All EDs use the rapid emergency triage and treatment system (RETTS^11^) that includes five priority levels: Highest priority 1 (Red); Priority 2 (Orange); Priority 3 (Yellow); Lowest priority 4 (Green); and Priority primary care (Blue). The RETTS set of chief complaints are thus common for all EDs in the SEM cohort. All EDs have similar access to patient testing, and clinical guidelines are generally the same in the entire region.

During and after the data collection period, the patients were informed of the purpose and structure of the SEM cohort in writing via public advertising on a website, and that they could decline participation at any time, for any reason, by contacting a research nurse or the first author at Lund. The creation of the SEM cohort and its use for AI/ML research and cross-sectional analyses has been approved by the Swedish Ethical Review Authority (Dnr 2019-05783), and by Region Skåne (302-19). There is no approval for commercial use of the data.

*Data collection*

During the study period, all patients at the eight EDs were included in the SEM cohort by default via identification in the common ED patient log system (Patientliggaren,^Tm^ Tietoevry^12^), and data from the other registers (below) were then linked by each patient’s unique Swedish identification (ID) number, which is universally used in Swedish healthcare and all government registers. After collection and linkage, all data were pseudonymized with patient study ID numbers and kept on secure servers behind firewalls at Lund University where access is logged. The key between personal and study IDs is kept separately on a Region Skåne server with standard healthcare data security.

The data sources include healthcare databases and registers with complete national or regional coverage, which should ensure close to complete data on all patient visits. As much as possible, we used well described high-quality data sources (see e.g. references^13-16^) to collect the SEM data in order to decrease bias and data errors. The number of missing data varies across the sources but is generally very low. Data variables were chosen based on importance in the emergency care process as well as availability in the source registers. The collected data were the same as used in clinical care, and there was no major change in data labelling during 2017-2018. The SEM cohort was not designed with a specific CDSS or study in mind, but the size of the cohort (below) and the number of variables and data included was chosen to ensure sufficient statistical power for most CDSS research projects.

Data from the source registers were kept in their exported form with no deletion or curation, and software scripts are used to extract data to form tailor-made new datasets for each specific research project. Data curation or deletion will generally take place in each CDSS project, and only as needed in the original SEM cohort data.

As shown in Table 2, the available data for each patient visit include the patient’s baseline data, data on the ED visit, and the outcome within 30 days up to three years after the ED visit: diagnoses, ED returns, hospital admissions, death, and healthcare costs. In total, the SEM data include several hundred variables for each patient, and many more that can be calculated from the original variables, such as ED crowding or boarding data, return visits, and mortality at different times after ED arrival. Detailed variable lists are available on reasonable request.

The SEM cohort is thus mainly based on register data and does not include free text information such as the patient’s detailed symptom history, findings at the physical examination, reasons for decisions and preliminary assessments. Also missing are the initial ED vital signs (blood oxygen saturation, respiratory rate, pulse rate, blood pressure, consciousness level and body temperature) and pharmacological treatment in the ED, since these data are primarily recorded on paper in the region. However, all this missing information can be obtained as needed by manual review of the individual patient records. As for diagnostic tests, ECG data are available as the raw signal, amplitude/interval measurements as well as the machine interpretation, and imaging and functional test data are available as the free text results. The images are not part of the SEM cohort data but can be obtained in specific projects.

*Basic cohort characteristics*

The SEM cohort is briefly described in Table 3 and includes 325 539 unique patients with 630 275 ED visits during 2017 and 2018. At the visit, fewer than five patients declined participation which makes the cohort almost 100% complete. The mean age was 55 years, 49% were male and 23.5% of all patients arrived by ambulance. The most common triage category was 3, Yellow, and 15.0% of the patients had no registered triage category mostly due to immediate referral from the ED to external primary care or self-care. Eleven percent of the patients had previous diagnoses of diabetes, 10% of cancer, 8% of pulmonary disease, and 1.7% suffered from dementia.

Table 4 shows that the most common chief complaint in SEM was abdominal pain, followed by chest pain, dyspnea, hand injury and unspecific disorder. (The term “unspecific disorder” is used when the triage nurse is unable to classify the patient’s problem using the more specific terms in the system.) Some 9% of all visits had no registered chief complaint, again mostly because of immediate referral to primary or self-care. The median time to doctor was 70 min and the median length of stay was 206 min. In 24% percent of all ED visits the patient was admitted to in-hospital care.

As can be seen in Table 5, the most common discharge diagnoses were bacterial pneumonia, cerebrovascular incident, and acute myocardial infarction. The mortality at the ED was 0.2%, it was 0.9% within 7 days, and 2.2% within 30 days.

**Discussion**

In addition to CDSS development, SEM’s large amount of real-world ED patient data with almost complete follow-up will allow research in many fields of emergency medicine: Epidemiology, patient management, diagnostics, prognostics, ED crowding, resource allocation, and social medicine. Some of these studies may need supplementary ethics approval. The SEM cohort is currently being used to analyze cases of missed acute aortic syndrome, for prediction of venous thromboembolism, mapping of characteristics and outcomes in patients with dizziness or with head trauma, and for the evaluation of emergency care for adult patients with congenital heart disease.

Studies of the epidemiology of ED patients may be beneficial for public health surveillance, resource planning, evaluating healthcare delivery and for facilitating research, e.g. sample size calculations for prospective studies. Epidemiological information supports clinical evidence-based decision-making and enables the ED to organize according to the needs of the population. The SEM cohort includes almost all patients presenting at eight EDs in southern Sweden during two years, and it should therefore be possible to obtain reasonably accurate and generalizable data on chief complaints and underlying disease states in the entire population as well as in subgroups based on age, sex, comorbidities or sociodemographics. Also, diurnal, weekly, and seasonal variations may be described.

ED patient management and its impact on outcomes may be studied in the SEM cohort by analyzing e.g. waiting times, length of ED stay, admissions to intensive care, as well as patients who left without being seen by a physician or who returned to the ED. These analyses may also be made in the absence or presence of ED crowding. As mentioned, pharmaceutical treatment at the ED is not immediately available but can be extracted for all patients from the digitized (scanned) ED patient paper records.

The SEM cohort allows analysis of the accuracy of diagnostic and functional testing by comparing pre-test probability with short or medium-term outcomes such as specific diagnoses or death.

The utilization and costs of diagnostic testing, hospital admission and care at specific wards in each patient up to 30 days in the cohort can be used to analyze resource use in all patients and in specific subgroups. Also, the SEM cohort may be used to evaluate ED care and acute healthcare consumption in different socioeconomic and demographic groups, as well as inequalities and possible discrimination.

***Strengths and limitations***

SEM includes real-world clinical data from consecutive patients presenting to eight different EDs during two years. The large number of patient visits, variables, and clinical events should be sufficient for most analyses of interest. Data were collected in regular care and there are several general advantages with using routine care data when building CDSS. Firstly, it provides access to large amounts of data from a diverse and unselected patient population, which is crucial for developing CDSS that work across different patient demographics. Secondly, routine care data may be immediately available, reducing the cost and time required to collect data. Finally, routine care data collection will often allow simple tracking of patient outcomes and evaluation of the effectiveness of the CDSS, especially in a country with comprehensive healthcare databases like Sweden. In the future, it may be possible to use native, uncurated electronic health records directly for medical research.^17^ Another strength of the multimodal SEM cohort is its potential utility in developing CDSS that provide relative risks of multiple diagnoses, in contrast to algorithms based on a single type of input and output (e.g. radiology algorithms detecting cancer), and current clinical decision support tools which often serve merely as rule-out tests, e.g. the PERC rule for pulmonary embolism.

SEM includes data from ED patient visits in one Swedish region, and the data may therefore not be generalizable to other populations or healthcare settings. There are few patients in the SEM cohort with problems related to psychiatry, obstetrics/gynecology, and ophthalmology, as well as few pediatric patients without orthopedic problems. Some clinical variables are missing or less readily available in SEM, e.g. free text imaging results that require manual review, and this will of course prevent or complicate the creation of some types of CDSS, as well as some data disaggregation. Missing data in SEM are rare, but there may of course be errors in the data, which can lead to biased or inaccurate CDSS. Since SEM data were registered as part of regular care, bias may also arise from different patient evaluation and management based on previous clinical findings (verification bias) or based on patients’ ethnic or socioeconomic background. Also, historical bias will exist in any clinical database, i.e. when the data no longer accurately reflect a new healthcare reality.

Several variables in the SEM database were originally manually entered or determined subjectively, such as time stamps in the ED and discharge diagnoses and may therefore contain errors or bias. Diagnoses might also have been registered several times for the same care episode. Bias or errors in the training data will cause a high risk of bias in the final CDSS, but the size and impact of the problem will vary in different CDSS. The optimal approach to the potential problem with bias is therefore best determined in each use case and CDSS. Before clinical implementation, any CDSS based on SEM data should be carefully reviewed and prospectively tested in a clinical trial in the specific healthcare setting.

On the other hand, it should be noted that if a CDSS is intended to operate in real time with standard register data as input, it is preferable that the underlying ML model is developed using this type of data rather than curated data that do not reflect the “dirty” truth of day-to-day operations. With sufficiently large training data, current ML algorithms can cope with a fair amount of noise and navigate between varying levels of noise in different types of input data.

In addition to algorithm quality, several barriers to successful implementation and use of AI/ML-based CDSS must be considered: IT problems, low model transparency (black box algorithms), proprietary code, lack of trust and knowledge among physicians and decision-makers, legal framework (oversight, malpractice issues) and ethical issues, integrity risks and financial challenges.^18-20^ However, the size and implications of these barriers will vary in different use cases.

In conclusion, the SEM cohort provides a platform for collaborative CDSS research. SEM’s large amount of real-world patient data with almost complete follow-up will also allow research in epidemiology, patient management, diagnostics, prognostics, ED crowding, resource allocation, and social medicine.

#### SEM cohort Access

So far, collaborations have been established with other research groups at Lund and Halmstad Universities in Sweden. We welcome initiatives on international collaborative projects using the SEM cohort. Anonymized parts of the SEM database will be available for sharing on reasonable request, as will detailed variable lists. Please contact the corresponding author via email ([ulf.ekelund@med.lu.se](mailto:ulf.ekelund@med.lu.se)).

**declarations**

*Ethics approval and consent to participate*

The creation of the SEM cohort and its use for AI/ML research and cross-sectional analyses has been approved by the Swedish Ethical Review Authority (Dnr 2019-05783) and Region Skåne (KVB 302-19). There is no approval for commercial use of the data. All included patients had access to written information on the SEM cohort and its purpose, and had the possibility to decline participation at any time, for any reason.

*Consent for publication*

Not applicable

*Availability of data and materials*

Anonymized parts of the SEM database will be available for sharing on reasonable request. Please send an email to [ulf.ekelund@med.lu.se](mailto:ulf.ekelund@med.lu.se).

*Competing interests*

None of the authors declare competing interests

*Funding*

The study was supported by an ALF research grant at Skåne University Hospital and by a grant from Region Skåne. This study was part of the AIR Lund (Artiﬁcially Intelligent use of Registers at Lund University) research environment and received funding from the Swedish Research Council (VR; grant no. 2019-00198). There was no industry involvement. Funding organizations had no role in the planning, design, or conduct of the study, collection, analysis or interpretation of data, or preparation, review or approval of the manuscript.

*Author contributions*

UE, BO, and OM conceived the cohort, were responsible for the ethics approval, and generated funding together with JB. JB, MO, JLF and POC provided expert opinion in the design of the cohort and the database. AB led the data management together with AN and made the general data analyses. UE and AB drafted the manuscript. All authors critically revised and approved the final manuscript and meet the criteria for authorship established by the ICMJE.

*Acknowledgements*

We are grateful for the excellent help and project coordination by Cecilia Åkesson Kotsaris, and for the invaluable data management by Paul Söderholm, both at Region Skåne. We also thank the patients for their participation, and the research assistants and the emergency department staff in Region Skåne for their kind help.

**Table 1. Characteristics of the EDs included in the SEM cohort**, after Welch et al.^21^

*trauma level according the American College of Surgeons.^22^ EM, emergency medicine; ENT, Ear nose and throat; Ob/Gyn, Obstetrics/Gynecology

| **Hospital** | **Number of patient visits in SEMD** | **Admission rate, %** | **Trauma level*** | **Specialties present (patient spectrum received)** | **Open 24/7/365** | **Transplant Service in hospital** | ***Acuity*** | **EM specialist training program** |
| --- | --- | --- | --- | --- | --- | --- | --- | --- |
| **Malmö** | 157 825 | 25 | 2 | Internal Medicine, Neurology, Surgery, Urology, Orthopedics & Trauma, Infectious diseases, Pediatrics, ENT | *Yes* | *Yes* | *High* | *Yes* |
| **Helsingborg** | 141 621 | 20 | 2 | Internal Medicine, Neurology, Surgery, Urology, Orthopedics & Trauma, Infectious diseases, Pediatrics, ENT, Urgent primary care, Ophtalmology | *Yes* | *No* | *High* | *Yes* |
| **Lund** | 128 851 | 26 | 1 | Internal Medicine, Neurology, Surgery, Urology, Orthopedics & Trauma, Infectious diseases, ENT | *Yes* | *Yes* | *High* | *Yes* |
| **Kristianstad** | 95 690 | 24 | 2 | Internal Medicine, Neurology, Surgery, Urology, Orthopedics & Trauma, Infectious diseases, Pediatrics, Ob/Gyn, ENT | *Yes* | *No* | *High* | *Yes* |
| **Ystad** | 52 078 | 30 | 3 | Internal Medicine, Neurology, Surgery, Urology, Orthopedics & Trauma, Infectious diseases, Pediatrics, ENT, Ob/Gyn, Ophtalmology | *Yes* | *No* | *High* | *Yes* |
| **Trelleborg** | 24 704 | 25 | 3 | Internal Medicine, Neurology, Surgery, Urology, Orthopedics & Trauma, Infectious diseases | *No* | *No* | *High* | *No* |
| **Hässleholm** | 18 905 | 32 | No trauma patients | Internal Medicine, Neurology | *No* | *No* | *Low* | *No* |
| **Landskrona** | 10 210 | 32 | No trauma patients | Internal Medicine | *No* | *No* | *Low* | *No* |

**Table 2. Available data for each patient visit in the SEM cohort.**

| ***Type of data*** | ***Description and examples of variables/ variable groups*** | ***Source*** |
| --- | --- | --- |
| ***Baseline data*** | | |
| Basic and sociodemographic patient characteristics | Age, sex, country of birth, education, marital status, place of residence, income, welfare benefits etc | 1 |
| Previous disease | Diagnoses (ICD10 codes) in the region during 5 years before ED visit | 8 |
| Previous tests | Diagnostic/ prognostic tests in the region during 5 years before the ED visit | 8, 9, 10, 11, 12 |
| Current medication | Prescriptions redeemed in Sweden during one year before ED visit | 2 |
| ***Data on the ED visit*** | | |
| Chief complaint | As defined by the RETTS system^11^ | 7 |
| ED throughput times | ED arrival time and date, time to ED nurse, ED physician, ED length of stay, ED discharge time and date. | 6, 7 |
| Findings at the physical examination, vital signs | Free text, available by manual review of the patient records | 8 |
| Medications | Given at the ED (free text) and after the ED but within 24h after ED presentation (digitized) | 8 |
| Diagnostic/prognostics tests | Performed within 24h after ED presentation, including ECG, imaging, functional tests etc, with free text results | 8, 9, 10, 11, 12 |
| Disposition | Left without being seen, admission to in-hospital care. | 7 |
| Preliminary diagnosis/ assessment at the ED | ICD10 codes and free text | 8 |
| ***Outcome data*** | | |
| In-hospital admission ward | Name (and type) of in-hospital ward | 8 |
| Hospital length of stay |  | 8 |
| Intensive care | Intensive care admissions/interventions within 30 days after ED presentation | 8 |
| Diagnoses | To the end of 2019 | 8 |
| Critical interventions/ treatments | Cardiac defibrillation, thrombolysis, percutaneous coronary intervention etc to end of 2019 | 8 |
| ED returns | Re-presentations at ED up to end of 2019 | 7 |
| Hospital admissions | Admissions to in-hospital care and length of stay to end of 2019 | 3, 8 |
| Date of death | To end of 2019 | 4, 6 |
| Cause of death | To end of 2019 | 5 |
| Healthcare costs | Direct healthcare costs in the region within 30 days after the ED visit. | 13 |

**SEM data sources**

*Registers and databases with national (Sweden) coverage*

1. Statistics Sweden;^13^ The LISA database, the Geography database

2. National Board of Health and Welfare; National Prescribed Drug Register^15,23^

3. National Board of Health and Welfare; National Patient Register; ^14,24^

4. The Swedish population register^13^

5. National Board of Health and Welfare; National Cause of Death Register^16,25^

6. Swedish emergency care register; SVAR^26-28^

*Region Skåne registers and databases with complete regional coverage*

7. The ED patient log system (Patientliggaren^Tm^, Tietoevry^12^)

8. The electronic healthcare records (Melior^Tm^, Siemens^29^) and/or the Patient administrative system (PASIS)

9. The ECG-database (MUSE^Tm^, GE Healthcare^30^)

10. The imaging and functional testing database (SECTRA^31^)

11. The clinical chemistry database (LIMS RS)

12. The microbiology database

13. The healthcare economics system

**Table 3. Baseline patient characteristics and management in the SEM cohort.**

Std, standard deviation. *Among the unique patients

|  |  |
| --- | --- |
| Patient visits, n | 630275 |
| Unique patients, n | 325539 |
| Male, n, % | 308990, 49.0% |
| Age, mean (std) | 55.2 (21.8) |
| Arrival by ambulance, n, % | 147976, 23.5% |
|  |  |
| **Triage category** |  |
| Highest priority; 1. Red | 5.8% |
| Priority 2. Orange | 20.4% |
| Priority 3. Yellow | 46.4% |
| Lowest priority; 4. Green | 6.3% |
| Primary care, Blue | 6.1% |
| Missing | 15.0% |
|  |  |
| Time to doctor in min, median (iqr) | 70.0 (32.0-137.0) |
| ED length of stay in min, median (iqr) | 206.0 (99.0-346.0) |
|  |  |
| ECG registered | 29.4% |
| Imaging performed within 24h | 41.4% |
| Admitted to in-hospital care | 24.4% |
| ED revisit in 7d | 13.8% |
|  |  |
| **Previous diagnoses (ICD10 codes) at ED presentation** | **Prevalence*** |
| Diabetes (E109, E119, E139, E149, E101, E111, E131, E141, E105, E115, E135, E145) | 11.0% |
| Cancer (C0-3, C40-49, C5-6, C70-76, C80-85, C883, C887, C889-901, C91-93, C940-943, C9451, C947, C95-96) | 10.3% |
| Pulmonary disease (J40-47, J60-67) | 8.2% |
| Cerebrovascular incident (I60-63, I65-66, G450-452, G454, G458-459, G46, I64, I670-672, I674 I675-679, I681-682 I688, I69) | 7.3% |
| Congestive heart failure (I50) | 7.0% |
| Renal disease (N01, N03, N052-056, N072-074, N18-19, N25) | 4.0% |
| Peripheral vascular disease (I71, I790, I739, R02, Z958, Z959) | 2.6% |
| Dementia (F00-02, F051) | 1.7% |

**Table 4. Twenty most common chief complaints in the SEM cohort**, according to the RETTS system.^11^ The term “Unspecific disorder” is used when the triage nurse is unable to classify the patient’s problem using the more specific terms in the system.

|  | **Number of patient visits** | **% of all patients** |
| --- | --- | --- |
| Abdominal pain | 67171 | 10.7% |
| Missing | 57462 | 9.1% |
| Chest pain | 51351 | 8.1% |
| Dyspnea | 38154 | 6.1% |
| Injury, hand | 24507 | 3.9% |
| Unspecific disorder | 23302 | 3.7% |
| Extremity pain | 19814 | 3.1% |
| Injury, head | 18932 | 3.0% |
| Injury foot | 16255 | 2.6% |
| Dizziness | 15767 | 2.5% |
| Infection | 15451 | 2.5% |
| Arrhythmia | 14350 | 2.3% |
| Neurological deficit | 13498 | 2.1% |
| Fever | 12945 | 2.1% |
| Headache | 12288 | 1.9% |
| Back pain | 11622 | 1.8% |
| Extremity symptom | 10905 | 1.7% |
| Renal colic | 9939 | 1.6% |
| Injury, knee | 9566 | 1.5% |

**Table 5. Selected discharge diagnoses from the ED or from in-hospital care directly following the ED visit, in the SEM cohort.**

| **Discharge diagnoses** | **ICD-10 codes** | **n, % of all patients** |
| --- | --- | --- |
| Bacterial Pneumonia | J13-15, J18 | 17047, 2.7% |
| Cerebrovascular incident | I60-63, I65-66, G450-452, G454, G458-459, G46, I64, I670-672, I674 I675-679, I681-682 I688, I69 | 15400, 2.4% |
| Acute myocardial infarction | I21, I22 | 14258, 2.3% |
| Sepsis | A021, A207, A227, A392, A327, A394, A40, A41, R572, R65 | 6589, 1.0% |
| Intoxication | F100, F110, F120, F130, F140, F150, F160, F170, F180, F190 | 4851, 0.8% |
| Hip Fracture | S720-722 | 4267, 0.7% |
| Ileus | K56 | 3400, 0.5% |
| Pulmonary Embolism | I26 | 3113, 0.5% |
| Cholecystitis | K800-801, K804, K81 | 2955, 0.5% |
| Acute Appendicitis | K35, K36, K37 | 2623, 0.4% |
| Pancreatitis | K85 | 1759, 0.3% |
| Aortic Dissection | I710 | 204, 0.03% |
| **Death** | | |
| Death at the ED | | 1004, 0.2% |
| Death within 7 days of ED arrival | | 5969, 0.9% |
| 30 days | | 13755, 2.2% |
| 365 days | | 50908, 8.1% |

**References**

1. Muth C, Blom JW, Smith SM, et al. Evidence supporting the best clinical management of patients with multimorbidity and polypharmacy: a systematic guideline review and expert consensus. J Intern Med 2019;285(3):272-288. DOI: 10.1111/joim.12842.

2. Socialstyrelsen. Väntetider och patientflöden på akutmottagningar. 2015:1-80. (<http://www.socialstyrelsen.se/Lists/Artikelkatalog/Attachments/19990/2015-12-11.pdf>).

3. Lynch S. Andrew Ng: Why AI Is the New Electricity. Stanford Graduate School of Business. (<https://www.gsb.stanford.edu/insights/andrew-ng-why-ai-new-electricity>).

4. Obermeyer Z, Emanuel EJ. Predicting the Future - Big Data, Machine Learning, and Clinical Medicine. N Engl J Med 2016;375(13):1216-9. DOI: 10.1056/NEJMp1606181.

5. Tang X. The role of artificial intelligence in medical imaging research. BJR Open 2020;2(1):20190031. DOI: 10.1259/bjro.20190031.

6. Harrou F, Dairi A, Kadri F, Sun Y. Forecasting emergency department overcrowding: A deep learning framework. Chaos Solitons Fractals 2020;139:110247. DOI: 10.1016/j.chaos.2020.110247.

7. Sudarshan VK, Brabrand M, Range TM, Wiil UK. Performance evaluation of Emergency Department patient arrivals forecasting models by including meteorological and calendar information: A comparative study. Comput Biol Med 2021;135:104541. DOI: 10.1016/j.compbiomed.2021.104541.

8. NHS using AI to reduce ‘avoidable’ hospital admissions this winter. The Independent: <https://www.independent.co.uk/>; Nov 14, 2023.

9. Socialstyrelsen. Digitala vårdtjänster och artificiell intelligens i hälso- och sjukvården. Swedish National Board of Health and Welfare (Socialstyrelsen); 2019.

10. AIR Lund – Artificially Intelligent use of Registers at Lund University. Lund University. (<https://portal.research.lu.se/en/projects/air-lund-artificially-intelligent-use-of-registers> ).

11. Predicare. The RETTS system. (<https://predicare.com/>).

12. TietoEvry. Patientliggaren. (<https://www.tietoevry.com/>).

13. Statistics Sweden. (<https://www.scb.se/en/>).

14. Ludvigsson JF, Andersson E, Ekbom A, et al. External review and validation of the Swedish national inpatient register. BMC Public Health 2011;11:450. DOI: 10.1186/1471-2458-11-450.

15. Wettermark B, Hammar N, Fored CM, et al. The new Swedish Prescribed Drug Register--opportunities for pharmacoepidemiological research and experience from the first six months. Pharmacoepidemiol Drug Saf 2007;16(7):726-35. DOI: 10.1002/pds.1294.

16. Brooke HL, Talback M, Hornblad J, et al. The Swedish cause of death register. Eur J Epidemiol 2017;32(9):765-773. DOI: 10.1007/s10654-017-0316-1.

17. Khan MS, Usman MS, Talha KM, et al. Leveraging electronic health records to streamline the conduct of cardiovascular clinical trials. Eur Heart J 2023. DOI: 10.1093/eurheartj/ehad171.

18. Stewart J, Lu J, Goudie A, et al. Applications of machine learning to undifferentiated chest pain in the emergency department: A systematic review. PLoS One 2021;16(8):e0252612. DOI: 10.1371/journal.pone.0252612.

19. Panch T, Mattie H, Celi LA. The "inconvenient truth" about AI in healthcare. NPJ Digit Med 2019;2:77. DOI: 10.1038/s41746-019-0155-4.

20. Goodman KE, Rodman AM, Morgan DJ. Preparing Physicians for the Clinical Algorithm Era. N Engl J Med 2023;389(6):483-487. DOI: 10.1056/NEJMp2304839.

21. Welch S, Augustine J, Camargo CA, Jr., Reese C. Emergency department performance measures and benchmarking summit. Acad Emerg Med 2006;13(10):1074-80. (In eng). DOI: j.aem.2006.05.026 [pii] 10.1197/j.aem.2006.05.026.

22. The American College of Surgeons CoT. Resources for Optimal Care of the Injured Patient: The American College of Surgeons, 2006.

23. National Prescribed Drug Register. National Board of Health and Wellfare. Jan 14, 2022 (<https://www.socialstyrelsen.se/en/statistics-and-data/registers/national-prescribed-drug-register/>).

24. National Patient Register, Swedish National Board of Health and Welfare. (<https://www.socialstyrelsen.se/en/statistics-and-data/registers/national-patient-register/>).

25. National Cause of Death Register. National Board of Health and Welfare. (<https://www.socialstyrelsen.se/en/statistics-and-data/registers/>).

26. Letterstal A, Ekelund U, Castren M, Lindmarker P, Safwenberg U, Kurland L. [SVAR--a unique Swedish emergency registry]. Lakartidningen 2010;107(43):2659-60. (<http://www.ncbi.nlm.nih.gov/pubmed/21137537>).

27. Ekelund U, Kurland L, Eklund F, et al. Patient throughput times and inflow patterns in Swedish emergency departments. A basis for ANSWER, A National SWedish Emergency Registry. Scand J Trauma Resusc Emerg Med 2011;19:37. DOI: 10.1186/1757-7241-19-37.

28. The Swedish Emergency Care Registry. SVAR. (<https://www.ucr.uu.se/svar/>).

29. Siemens Healthineers. (<https://www.siemens-healthineers.com/en-uk>).

30. Healthcare G. (<https://www.gehealthcare.com/>).

31. SECTRA. (<https://sectra.com/>).
