# Supplementary material for: The skåne emergency medicine (SEM) cohort
Source: Scand J Trauma Resusc Emerg Med. 2024 Apr 26;32:37. doi: 10.1186/s13049-024-01206-0 (PMC11046860; doi:10.1186/s13049-024-01206-0)
Supplement: Supplementary file 1 — Supplementary Material 1 [file 13049_2024_1206_MOESM1_ESM.docx]

# Dear Editor,

Date 2024-02-10

Please find enclosed our revised manuscript with the title ”The Skåne Emergency Medicine (SEM) cohort” and reference number STRE-D-23-00349R1.

We are grateful for the thoughtful and thorough comments by the reviewer and have made all the suggested changes. In addition to this, we have made very minor alterations to improve language and logic. We are convinced that the manuscript has been improved.

Our answers to the comments and recommendations by the Reviewer are described point-by-point on the following pages. See also the marked new version of the manuscript.

It is our hope that the manuscript can now be accepted for publication in Scandinavian Journal of Trauma, Resuscitation and Emergency Medicine.

Sincerely

Professor Ulf Ekelund

Emergency Medicine

Lund University

***Replies to reviewers***

***Reviewer #1***

***Line 5 would suggest changing to "this has increased steadily year-on-year by by 2-3%"***

Reply: This has been changed according to the suggestion.

***Line 26 would suggest changing to "their chief complaint"***

Reply: As suggested, we have inserted the word ”their”

***Line 29 would suggest changing to "form of subsequent diagnoses, treatments, healthcare costs and mortality within a follow-up period of at least 30 days, and up to 3 years"***

Reply: This has been changed according to the suggestion.

***Line 36 consider exchanging "resource" for "platform".***

Reply: In our version of the manuscript, the word “platform” is already used. If the reviewer suggests a change from “platform” to “resource”, we will be happy to make this change.

***Line 41 qualify "complete follow-up" with the duration of follow up (e.g. "short term follow up"), or include the follow-up duration in line 29***

Reply: As suggested, we have inserted the words “short-term”.

***P4***

***Line 12 would suggest changing to "outcomes"***

Reply: This has been changed according to the suggestion.

***Line 14 "exploding" - consider replacing with "also increasing"***

Reply: This has been changed according to the suggestion.

***Line 17 qualify with "Often the ED physician can no longer grasp and process all available information, making it impossibible for an individual clinician to provide the theoretically best possible care"***

Reply: This has been changed according to the suggestion.

***Line 41 This is an excellent point***

Reply: Thank you.

***Line 51 "describe the rationale for, and construction of, the Skane Emergency Medicine cohort"***

Reply: This has been changed according to the suggestion.

***P6***

***Line 17 How many hits were there on the website?***

Reply: This information is unfortunately not available to us.

***P7***

***Line 46 An excellent description of missing data - thank you***

Reply: Thank you.

***P9***

***Line 34 Replace Swedish with Sweden***

Reply: Thank you for noticing this simple error. “Sweden” is now used.

***P10***

***Line 51 Should mention the lack of paeds/obs/gynae pysch presentations when considering generalisability.***

Reply: In accordance with the suggestion, we have in the following line inserted the sentence:

“There are few patients in the SEM cohort with problems related to psychiatry, obstetrics/gynecology, and ophthalmology, as well as few pediatric patients without orthopedic problems.”

***P11***

***Line 5 Suggest including here a definition of historical bias***

Reply: As suggested, we have added a definition: “...when the data no longer accurately reflect a new healthcare reality.”

**Additional comments provided in the separate document (Attachment):**

For clarity, the authors’ replies below are in blue. All page references are to the new version of the manuscript.

***Background***

***Key topics which should be addressed in the introduction: consent, power, inclusivity,***

***transparency, ethics and privacy.***

***Reply: We agree that these topics are crucial. We have extensively revised the ms,***

***and in the new version, we deal these topics for the SEM cohort in Methods/Design:***

***Consent (P6, second para), inclusivity (Table 1 and last sentence on P5) power (P7,***

***end of first para), privacy (P6, last para) and ethics (Declarations and on P6, second***

***para). Transparency will of course be different for every CDSS, and we mention the***

***importance of this in Discussion (P11, end of second para).***

***We thus believe that these topics are better addressed in Methods/Design, but if the***

***Editor would like us to move them to Background, we will of course be happy to do so.***

***I agree that these topics are now well-covered in the methods. In my opinion, it would still be worthwhile to include a sentence at the end of the Background section, reflecting the fact that the MS now deals with these crucial topics (ethics, data protection, bias) and describes the process of building a dataset with careful consideration of these topics.***

Reply: As suggested, we have inserted a sentence at the end of Background:

”We describe in this paper the process of building the SEM dataset with careful consideration of ethics, data protection, and bias.”

***The first paragraph of the discussion should be included in the introduction, and used***

***to frame the "mission statement" of the dataset - in turn this makes it clear what data***

***would be needed in order to answer the questions sought, which in turn makes it clear***

***what the dataset is missing.***

***Reply: In the new version of the ms, we have moved the first paragraph of Discussion***

***to the Background and believe that this has improved the ms.***

***Concur.***

Reply: Thank you.

***No references presented for hyperbolic statements in lines 12-22***

***Reply: Good point. We have modified the text and provided references for all***

***statements. The text now reads:***

***“Artificial intelligence (AI) and machine learning (ML) are now developing fast, and***

***most industries will likely be fundamentally changed by AI in the coming years.3 In***

***medicine, AI and ML provide new possibilities when applied to extensive electronic***

***health records and registers.4 The most impressive advances have occurred in***

***radiology and pathology, where ML accuracy of image classifications now exceeds that***

***of humans.5 In emergency medicine, AI/ML-driven decision support tools have the***

***Powered by Editorial Manager® and ProduXion Manager® from Aries Systems Corporation***

***potential to improve diagnostic accuracy,5 alleviate ED crowding,6,7 and decrease the***

***use of inpatient beds and healthcare costs.8 ”***

***Concur***

Reply: Thank you.

***No critique is offered by the authors of the difference between AI providing low-stakes***

***suggestions in areas which are not evidence-based and have been difficult to predict***

***(i.e. consumer choices) and AI providing suggestions as to medical diagnoses which***

***are derived from strict deductive reasoning and an iterative process of hypothesis***

***testing. This is a critical distinction***

***Reply: We agree that this is a critical distinction and have removed the reference to***

***consumer choices (former ms version P4, line 11-12) in Background. The text will now***

***hopefully not lead the reader to believe that useful medical AI can be developed in the***

***same way as low-stakes applications, e.g. discounts in supermarkets.***

***Concur***

Reply: Thank you.

***No comment is made by the authors on the risks or limitations of ML algorithms (i.e.***

***training data needs to represent the real world; need to be vigilant about the scope,***

***quality and handling of data; biases in data will be reflected in algorithms), nor on the***

***potential ethical dilemmas caused by "black box algorithms"***

***Reply: These are all very important points. In the new version of the ms, we describe***

***that all SEM cohort data are from real-world care in Methods/Design, P7, first para,***

***lines 6-7:***

***“The collected data were the same as used in clinical care….”,***

***and Discussion, e.g. first sentence:***

***“SEM’s large amount of real-world ED patient data with almost complete follow-up…..”***

***Further, we mention the importance of data quality in Background, P4, end of second***

***para:***

***“The creation of ML-based decision support for ED use requires large amounts of high-***

***quality clinical data, preferably from representative unselected ED patients in routine***

***care.”***

***and we acknowledge the potential errors and biases in the data and CDSS in***

***Strengths and Limitations (P10, last para and P11, first and second para) as well as***

***the general problem of black box algorithms (Strengths and Limitations, P11, next to***

***last para).***

***We believe that a general and in-depth discussion of these problems is beyond the***

***scope of our paper, especially since the implications might vary in different use cases.***

***I agree that a general and in-depth discussion is beyond the scope of this paper, and think you raise an excellent point that the implications will vary in different use cases – I would suggest including a short sentence to this effect in the discussion, for example after p11 Line 51.***

Reply: Thank you for this comment. We already state on P11, last para that:

“Bias or errors in the training data will cause a high risk of bias in the final CDSS, but the size and impact of the problem will vary in different CDSS.”

As suggested, this has now been supplemented with a new sentence on P12, end of third para:

“However, the size and implications of these barriers will vary in different use cases.”

***Machine learning algorithms for image classification can outperform humans, but have***

***also been shown to overfit to artefact. Also, machine learning algorithms, trained on a***

***dataset with a single input (an image) and a single, defined output (presence or***

***absence of a disease) are much more amenable to testing (with alternate datasets,***

***and edge cases) than algorithms which consider a vast array of inputs and outputs***

***(such as in this dataset)***

***Reply: We completely agree but are unsure whether the reviewer wants us to include a***

***general discussion of this in the ms. If not, we plan to discuss the strengths and***

***limitations of the specific AI/ML models and CDSS developed using the SEM cohort in***

***future papers. (We note in passing, however, that there is a duality to the reviewers comment –***

***overfitting to artifacts in data is less likely when data is multi-modal and/or originate***

***from multiple disjoint sources. Moreover, although specialized ML-approaches may be***

***successful at binarily identifying presence or absence of a certain condition, this is***

***rarely the utility needed in ED care. For instance, if an ML tool can rule out AMI in a***

***patient presenting with chest pain this does not mean that the patient can be***

***discharged, as there may be other serious conditions requiring immediate treatment.***

***The SEM cohort dataset allows this complete view which can aid ML developers to***

***create algorithms discriminating between, e.g., AMI, aortic dissection, or pulmonary***

***embolism using a variety of input data.)***

***Thank you for your thoughtful response to my comment: I agree that discussing strengths/weakness of specific ML/AI models is beyond the remit of this MS. However, I think that the more general point is an interesting one which merits inclusion in the discussion: I would like to see a line differentiating between the multimodal SEM cohort, and its potential utility in developing tools which weigh up relative risks of multiple diagnoses and a) ML algorithms based on a single type of input and output (e.g. radiology algorithsm), b) current clinical decision tools and guidelines which typically work on the basis of clinicians performing the Bayesian analysis and the tool serving merely as a rule out test e.g. PERC score.***

Reply: As suggested, we have added the following on P11, first para:

“Another strength of the multimodal SEM cohort is its potential utility in developing CDSS that provide relative risks of multiple diagnoses, in contrast to algorithms based on a single type of input and output (e.g. radiology algorithms detecting cancer), and current clinical decision support tools which often serve merely as rule-out tests, e.g. the PERC rule for pulmonary embolism.”

***No consideration given to why there have been no ML/AI algorithms used in routine ED***

***care: what are the barriers to their safe and equitable implementation.***

***Reply: Thank you for this comment. We have added the following at the end of***

***Strengths and Limitations (P11, next to last para), and believe that this has improved***

***the ms:***

***“In addition to algorithm quality, several barriers to successful implementation and use***

***of AI/ML-based CDSS must be considered: IT problems, low model transparency***

***(black box algorithms), proprietary code, lack of trust and knowledge among physicians and decision-makers, legal framework (oversight, malpractice issues) and ethical issues, integrity risks and financial challenges.18-20 ”***

***Thank you for this inclusion, I think it adds considerably to the discussion.***

Reply: Thank you.

***No mention made of the key issue here, which is dataset quality. It is critical to report***

***and document the subjective judgments and biases and contingent contexts involved***

***in dataset production in order to offset later problems with bias.***

***Reply: Indeed, this is a key issue. In the revised ms, data quality in the SEM cohort is***

***now described in Methods/Design (P7, first and last paras) and discussed in Strengths***

***and Limitations (P10, last para, and P11, first and second para).***

***We have also added on P11, third para, that:***

***“On the other hand, it should be noted that if a CDDS is intended to operate in real time***

***with standard register data as input, it is preferable that the underlying ML model is***

***developed using this type of data rather than curated data that do not reflect the “dirty”***

***truth of day-to-day operations. With sufficiently large training data, current ML***

***algorithms can cope with a fair amount of noise and navigate between varying levels of***

***noise in different types of input data.”***

***Thank you for these excellent additions which I think meaningfully characterise the risks of bias, and the benefits and risks of using “real” clinical data – these are of course the “dirty data” that clinicians use to make their decisions. I think including a definition of historical bias (p11 line 5) would be useful for readers who are not familiar with ML/AI – it is a key concept.***

Reply: As described above, we have added a short definition on p11, end of second para:

“…when the data no longer accurately reflect a new healthcare reality.”

***This publication does not make any comment on the fact that the characteristics of a***

***training dataset influence the model's behaviour, and that bias in the dataset produces***

***models that replicate that bias.***

***Reply: We now mention this on P11, second para:***

***“Bias or errors in the training data will cause a high risk of bias in the final CDSS….”***

***A publication about a dataset should therefore be focussed on:***

***Describing the process of dataset construction sufficiently (motivation, composition,***

***collection processes, and recommended uses) such that readers, and users of the***

***dataset can understand the weaknesses of the dataset, and the representational and***

***historical biases within it***

***Reply: We have revised the text extensively and now cover the motivation of the SEM***

***cohort (last para of Background), the composition (Table 2 and Methods/Design),***

***collection processes (Data collection, P6-8), and recommended uses (Discussion, first***

***part up to Strengths and limitations).***

***Agreed. Revisions are excellent.***

Reply: Thank you.

***What are the errors, sources of noise, redundancies or inter-related relationships within***

***the dataset***

***Reply: General errors and sources of noise, as well as an obvious redundancy***

***(diagnoses appearing several times in the patient records and thus in the SEM data),***

***are described in Strengths and Limitations (P10-11). It is not clear to us what the***

***reviewer means by “inter-related relationships.” The pseudonomized SEM data are***

***linked by each patient’s study ID, as now stated on P6, last para:***

***“…and data from the other registers (below) were then linked by each patient’s unique***

***Swedish identification (ID) number, which is universally used in Swedish healthcare….”***

***Apologies for the lack of clarity – what I meant by inter-related relationships is that there are likely to be data points within the dataset which are highly correlated, and those correlations may be clinically meaningful, or meaningless. I think this is now adequately covered by your explanation of how the raw unredacted data are available when constructing CDSS.***

Reply: Thank you

***What key data are missing, and why. How might this bias the dataset.***

***Reply: Missing data points in the SEM cohort are few, as we state on P7, first para:***

***“The number of missing data will vary across the sources but are generally very few.”***

***Missing data in the variable “Triage category” are described in Table 3, and in the***

***variable “Chief complaint” in Table 4.***

***Regarding missing variables, we now describe the most important ones in***

***Methods/Design (P7, last para and P8, first para):***

***“The SEM cohort is thus mainly based on register data and does not include free text***

***information such as the patient’s detailed symptom history, findings at the physical***

***examination, reasons for decisions and preliminary assessments. Also missing are the***

***initial ED vital signs (blood oxygen saturation, respiratory rate, pulse rate, blood***

***pressure, consciousness level and body temperature) and pharmacological treatment***

***in the ED, since these data are primarily recorded on paper in the region. However, all***

***this missing information can be obtained as needed by manual review of the individual***

***patient records.”***

***We find it hard to describe how the missing variables will affect algorithm bias since it***

***depends on the specific use case and ML model. We therefore plan to discuss this in***

***depth in future manuscripts describing specific models and applications.***

***Thank you for this clarification – it makes it clear how the dataset can be interrogated and expanded (e.g. to include patient history) when building future CDSS. I do think it would be worth including a sentence in “limitations” about the fact that some data are easier to access and incorporate into tools than others: realistically, no-one is going to develop a tool which requires them to manually review >600,000 hand-written notes!***

Reply: This is a very good point – indeed, there will not be a manual review of 600k notes. As suggested, we have inserted a sentence on P11, second para:

“Some clinical variables are missing or less readily available in SEM, e.g. free text imaging results that require manual review, and this will of course prevent or complicate the creation of some types of CDSS, as well as some data disaggregation.”

***Describing the processes used to explore bias within the dataset - disaggregation;***

***dataset ablations; audits; or manual review? AFLITE algorithm (Sakaguchi et al) looks***

***for spurious cues in datasets which would make them prone to overfitting.***

***Swayamdipta et al describe a tool which tests how models interact with their datasets -***

***identifying regions which the model finds "hard to learn" which often correspond to***

***mislabelling.***

***Reply: We agree, these are very important points to consider when creating AI/ML***

***models, but we believe that they are somewhat outside the scope of the present ms,***

***and that such analyses for all SEM data might make the ms unduly long. The SEM***

***database will likely never be used for modelingop in its entirety, and the risk of bias will***

***thus depend on which variables are included in the CDSS. Our plan is to use the***

***abovementioned tools when creating specific AI/ML models based on parts of the***

***database in future projects. If the Editor allows, we would therefore like to leave out***

***analyses with these tools in the present manuscript.***

***Concur. I think your MS is now much clearer in stating that there are inherent biases within the dataset which will need to be explored in each specific use case.***

Reply: Thank you.

***Methods***

***Methods doesn't mention the process of dataset construction/design/collaboration***

***(ethical approval; legal approvals; how data were assembled - what common identifiers***

***were used across all datasets) - which would be of interest to other researchers***

***looking to construct a local dataset***

***Reply: We now briefly describe the process of dataset construction (P6, last para, and***

***P7, first and second para), common identifiers (P6, last para) and ethical/legal***

***approvals (Declarations, and P6, second para).***

***Thank you – these clarifications add enormous value in terms of describing how similar datasets can be safely constructed and establishing a baseline for best practice.***

Reply: Thank you.

***No description of the ethical considerations weighed up when constructing the dataset.***

***No mention of whether separate ethical approval would be needed to develop***

***algorithms, or what constraints are put on the use of the data (e.g. could they be used***

***commercially?)***

***Reply: Good points. The ethics and legal approvals are described in Declaration and***

***on P6, second para, and we have now added that the ethics approval includes ML***

***model development, but not commercial use of the SEM cohort data:***

***“The SEM cohort and its use for AI/ML research and cross-sectional analyses has***

***been approved by the Swedish Ethical Review Authority (Dnr 2019-05783), and by***

***Region Skåne (302-19). There is no approval for commercial use of the data.”***

***Thank you.***

Reply: Thank you.

***Consent process not described in enough detail - how were people consented for this***

***use of their data; what was the opt-out process; can people opt out post facto?***

***Reply: We agree. The consent process, including that the patients could decline***

***participation at any time, is now described on P6, second para:***

***“During and after the data collection period, the patients were informed of the purpose***

***and structure of the SEM cohort in writing via public advertising on a website, and also***

***that they could decline participation at any time, for any reason, by contacting a***

***research nurse or the first author at Lund.”***

***Thank you. It would be good to include how many times that website was accessed as well as how many times people requested to be removed from the dataset: these are both important parameters when describing the efficacy of an opt-out process.***

Reply: Unfortunately, we have no data on the number of hits so far on the website, and since it includes general information about the study, it would be hard to know what the numbers would mean.

We do not know the exact number of patients who have opted out, and this is because it is not allowed to keep a list of these patients: they must be removed from the database and forgotten. The study website is still up, and patients will have the option to decline further participation for as long as the SEM database is used. So far, we estimate that less than 20 patients have opted out, but we are not sure how to interpret this. It seems likely that many patients have not seen the website, but on the other hand: Swedes are used to being registered everywhere and known for being docile and supportive of research. So, we find it hard to use this estimate as a measure of the effectiveness of the opt-out process. We believe that the opt-out process is reasonable and ethical, and all its details have been approved by the Swedish ethics review authority.

If acceptable to the Reviewer and the Editor, we would therefore like to not state the estimated number of patients who have declined participation so far.

***What protection is provided to prevent individuals from being identified from the***

***dataset?***

***Reply: Data protection is now described on P6, last para:***

***“After collection and linkage, all data were pseudonymized with patient study IDs and***

***kept on secure servers behind firewalls at Lund University where access is logged. The***

***key between personal and study IDs is kept separately on a Region Skåne server with***

***standard healthcare data security.”***

***Excellent description – thank you.***

Reply: Thank you.

***No description of why the datapoints included in the dataset were chosen - this is a key***

***factor in terms of:***

***Data disaggregation: if a datapoint is not included, it is not possible to disaggregate the***

***data on this point, and test for historical bias or representational bias, or test how well***

***algorithms work in that group***

***Determining whether the data are adequate to answer the clinical questions set***

***Reply: We fully agree. Regarding data choice, we now state on P7, first para, that:***

***“Data variables were chosen based on importance in the emergency care process as***

***well as availability in the source registers.”***

***Regarding disaggregation, we are unsure if the reviewer would like us to make***

***changes in the ms. We believe that a discussion of what projects that cannot be***

***performed using the SEM cohort data would make the ms unreasonably long.***

***I agree that it would not be feasible to exhaustively describe in the MS which projects would and would not be feasible. I think, however, that it would be sensible to incorporate into the discussion (perhaps around p11 line 27) a remark about how if a datapoint is not included, it cannot be a) included in the CDSS (which might seem obvious, but is an important consideration when designing a dataset, as datapoints which are obvious to a clinician (e.g. obesity) are not included in the CDSS if weight/BMI are not recorded… and weight/BMI might perversely be less likely be recorded in obese patients as clinicians may be concerned about causing offence) and b) used for data disaggregation.***

As suggested, we have inserted a sentence on P11, second para:

“Some clinical variables are missing or less readily available in SEM, e.g. free text imaging results that require manual review, and this will of course prevent or complicate the creation of some types of CDSS, as well as some data disaggregation.”

***What approach was taken to address and minimize biases within the dataset?***

***Reply: When collecting the data, we chose sources with as accurate and correct data***

***as possible, and this is now stated on P7, first para:***

***“As much as possible, we used well described high-quality data sources (see e.g.***

***references 13-16) to collect the SEM data in order to decrease bias and data errors.”***

***However, crude healthcare data will inevitably contain errors and/or bias. So the main***

***approach to this is identification, awareness, and consideration in the creation and***

***testing of each specific CDSS. This is now mentioned in Strengths and Limitations on***

***P11, second para:***

***“Bias or errors in the training data will cause a high risk of bias in the final CDSS, but***

***the size and impact of the problem will vary in different CDSS. The optimal approach to***

***the potential problem with bias is therefore best determined in each use case and***

***CDSS. Before clinical implementation, any CDSS based on SEM data should be***

***carefully reviewed and prospectively tested in a clinical trial in the specific healthcare***

***setting.”***

***Thank you for your inclusions of this important point.***

Reply: Thank you.

***Representational bias (divergence between true distribution of a characteristic, and its***

***distribution in the dataset) is not commented on. There is insufficient comment on the***

***data quality with respect to representational bias: 5th most common presentation was***

***"unspecific disorder", and no comment is made on this strange category: do patients***

***really attend the department and not specify what their concern is?***

***Reply: “Unspecific disorder” is a specified term in the RETTS triage system. Many***

***patients in emergency care cannot explain their problem, and when the triage nurse is***

***unable to classify the patient’s problem using the more specific terms in the system, he***

***or she will choose the term “unspecific disorder”. This type of unspecified chief***

***complaint exists in all triage systems for emergency care and does not represent***

***missing data.***

***We believe that the data in the SEM cohort database are very representative of the***

***patients currently presenting to the included EDs, and that representational bias is***

***therefore very low. However, it may of course not be representative of other***

***populations. We state on P10, last para, that:***

***“SEM includes data from ED patient visits in one Swedish region, and the data may***

***therefore not be generalizable to other populations or healthcare settings.”***

***Also, we write on P11, second para, that:***

***“…… before clinical implementation, any CDSS based on SEM data should be***

***carefully reviewed and prospectively tested in the specific healthcare setting.”***

***Thank you for clarifying the category of “unspecific disorder” – this category is described differently in different languages/systems (here, it is usually called “other”). I agree that the SEM data represent “real world” data and so including the category of “unspecified chief complaint” is useful, especially when developing CDSS which rely on this real world data. I think it would be useful to international readers to explain in the MS as you have done in your comment to me, that “when the triage nurse is unable to classify the patient’s problem using the more specific terms in the system, he or she will choose the term “unspecific disorder”.***

Reply: As suggested, we have inserted a sentence in the last para on P8:

“The term “unspecific disorder” is used when the triage nurse is unable to classify the patient’s problem using the more specific terms in the system.”

Also, we clarify this in the Table 4 legend:

“The term “unspecific disorder” is used when the triage nurse is unable to classify the patient’s problem using the more specific terms in the system.”

***My concern about representational bias is not that the SEM cohort would not be representative of ED patients, but rather that developers of CDSS need to consider whether the SEM dataset contain sufficient information to be representative of the ED population. E.g. all patients have a height and weight – is this documented in the SEM dataset? It would be easily guesstimated by a clinician at the bedside, but if it’s not in the dataset it won’t be in the CDSS.***

Reply: Ok, understood.

***What subpopulations are identified (e.g. age, gender) and how are they represented in***

***the dataset. What subpopulations are not represented***

***Reply: Since the SEM cohort includes data on all patients’ age and sex, their previous***

***diagnoses, their chief complaints and their diagnoses and outcomes, the number of***

***subpopulations possible to analyze is almost endless. However, some patient groups***

***are less common in the SEM cohort. We have now inserted a sentence on P5, last***

***para, to describe this:***

***“Patients with*** ***psychiatric disorders, problems related to obstetrics/ gynecology,***

***ophthalmology, and pediatric patients without orthopedic problems are few at all these***

***EDs, since there are specialized EDs for these patients in the region.”***

***We consider this a feature of the database, not a flaw.***

***Thank you for including this important clarification – it reflects the nature of the dataset, and has important implications for its generalisability. In terms of English syntax, the following phrasing would be more natural: “There are very few patients with psychiatric disorders, problems related to obstetrics/ gynecology, ophthalmology, and pediatric patients without orthopedic problems at these EDs, since there are specialised EDs for these patietns in the region.***

As suggested, we have now changed the sentence at the end of P5:

“There are very few patients with psychiatric disorders, problems related to obstetrics/ gynecology, ophthalmology, and pediatric patients without orthopedic problems at these EDs, since there are specialized EDs for these patients in the region.”

***Historical bias: replication of biased attitudes to testing and treatment***

***Reply: Good point. We now acknowledge the risk of historical bias with a new sentence***

***in Strengths and Limitations, P11, first para:***

***“Also, historical bias will exist in any clinical database and will have to be assessed and***

***considered in each use case and CDSS.”***

***Thank you. I repeat my previous comment that a definition of historical bias (either here, or where it is first mentioned) would be useful for the naive reader.***

Reply: This has been included as suggested, see reply above.

***Exclusion bias: what information has been deleted, or not included***

***Reply: We now state on P7, second para, that:***

***“Data from the source registers were kept in their exported form with no deletion or***

***curation…...”***

***Thank you***

Reply: Thank you.

***Measurement bias: was the data in the dataset collected in the same way as in the real***

***world, is the data in the dataset collected consistently across all sites (do all sites have***

***the same access to testing, the same clinical guidelines shopping their practice?)***

***Reply: Yes, all data are as collected in the same way as in routine care, and in the***

***same way at all sites. On P7, first para, lines 6-7, we now state that:***

***“The collected data were the same as used in clinical care.”***

***Also, on P6, first para, last lines, we now state that:***

***“All EDs have similar access to patient testing and clinical guidelines are generally the***

***same in the entire region.”***

***Thank you***

Reply: Thank you.

***Recall bias (are data labelled in similar ways across the dataset? If not, what are the***

***variables (patient related or hospital related or diagnosis related or clinician related)***

***which affect data labelling - these could become algorithmically important)***

***Reply: Yes, the data are labelled in similar ways in the entire dataset. We now state***

***this on P7, first para:***

***“… and there was no major change in data labelling during 2017-2018.”***

***Thank you***

Reply: Thank you.

***What data is missing?***

***Reply: See reply to the similar question above.***

***Thank you***

Reply: Thank you.

***The process for dealing with missing datapoints (and examining bias in missing data)***

***should be described.***

***Reply: The data sources are of high quality and missing data points are very few. In the***

***first para on P7, we now state that:***

***“The number of missing data will vary across the sources but is generally very low.”***

***We also now state in the second para on P7, that:***

***“Data from the source registers were kept in their exported form with no deletion or***

***curation, and software scripts are used to extract data to form tailor-made new***

***datasets for each specific research project.”***

***Because of the large number of variables, few missing data, and that the cohort data***

***will almost never be used in its entirety, we have chosen to defer the dealing with***

***missing data and potential bias to each specific CDSS project, were the importance***

***and optimal approach to these possible problems can be determined.***

***We now write on P11, second para:***

***“Bias or errors in the training data will cause a high risk of bias in the final CDSS, but***

***the size and impact of the problem will vary in different CDSS. The optimal approach to***

***the potential problem with bias is therefore best determined in each use case and***

***CDSS.”***

***Thank you***

Reply: Thank you.

***"Chief complaint" is missing in 9.1% of cases, and "unspecific disorder" is in 3.7% of***

***cases - how does this bias the data?***

***Reply: As mentioned in the ms (P8, second and last paras), most of these missing***

***chief complaint data appear in patients immediately discharged from ED after a very***

***brief evaluation. These patients will be of little interest in ML model creation. For***

***patients fully evaluated at the ED, almost no chief complaint data are missing.***

***As mentioned above, the chief complaint “Unspecific disorder” is universally used in***

***ED patients where the problem cannot be classified by the patient or the first ED***

***provider. It does not represent missing data.***

***Thank you – I refer to my previous comment about the clarification of “unspecific disorder”***

Reply: This has now been clarified in the manuscript, see reply above.

***The patient's history is documented, but how well? The documented history has***

***influenced which tests were ordered, but if it's not documented, then the algorithm can't***

***use that piece of information (although clinicians will use it as part of their decision***

***making process).***

***Reply: We have all the patient’s diagnoses in the 5 years before the ED presentation,***

***but we lack the exact symptom history of the patients, which is now stated on P7, last***

***para:***

***“…free text information such as the patient’s detailed symptom history…..is not***

***included.”***

***However, this information can be retrieved as needed for all patients by manual review***

***of the patient records which is stated on P8, second sentence.***

***Indeed, undocumented information will not be part of the SEM cohort data. The***

***importance of this will vary in different use cases, and we believe that a reasonable***

***approach is identification, assessment and consideration in the creation and testing of***

***the specific CDSS.***

***Agreed. I reiterate my previous comment that it would be beneficial to refer to the challenges of creating CDSS which rely on the detailed symptom history, due to the difficulties in retrieving this information manually.***

Reply: See reply above.

***There is no description of datapoints which are not included in the dataset, which***

***would be assumed to affect other datapoints (e.g. overcrowding in the department,***

***time of day at presentation are likely to affect "time to doctor").***

***Reply: We completely agree that crowding data and time of day is of importance for***

***provider decision-making, patient management, and patient outcome. In the SEM***

***cohort data, different crowding variables (e.g, number of patients present at the same***

***time, their combined waiting times and priority levels) can be calculated since all the***

***underlying data are available for all patients. This is now briefly mentioned on P7, third***

***para:***

***“….and many more that can be calculated from the original variables, such as ED***

***crowding or boarding data, return visits, and mortality at different times after ED***

***arrival.”***

***On P9, last para, we have added that:***

***“These analyses may also be made in the absence or presence of ED crowding.”***

***In Table 2, we have changed the middle column heading to clarify the content:***

***“Description and examples of variables/variable groups”***

***and we now also describe in this table that absolute ED arrival and discharge times***

***(and dates) for all patients are included in the SEM cohort data. Finally, we state on***

***P7, third para, that:***

***“Detailed variable lists are available on reasonable request.”***

***Thank you – this is an excellent addition.***

Reply: Thank you.

***Discussion***

***Generally fails to reference the key issues raised above***

***Reply: With the described changes in the ms, we believe that we cover all the key***

***issues raised above.***

***No reference made to bias in the dataset; or of the governance or ethical issues***

***around dataset creation***

***Reply: In the new version of the ms, we have added statements regarding the risks of***

***bias in the dataset (P10, last para, and P11, first and second para), and the ethical and***

***regional approvals are described in Methods/Design (P6, end of second para, and***

***Declarations). For details, see replies to the comments above.***

***Agreed***

Reply: Thank you.

***No reference made to the considerable issue of historical bias in the dataset,***

***particularly with the outcomes that the CDSS aims to predict (e.g. as regards inpatient***

***admission and ICU admission, both of which occur at clinician discretion)***

***Reply: We agree, historical bias should be mentioned. We have now inserted the***

***following in Strengths and Limitations on P11, first para:***

***“Also, historical bias will exist in any clinical database.”***

***Agreed – as previously I suggest a definition of historical bias should be included***

Reply: Thank you. A brief definition has been included in Discussion, see reply above.

***Artefacts in datasets result in models overfitting to the training data using illogical***

***shortcuts (decision rules which do not extrapolate well to data outside of the training***

***set) - how has the dataset been examined for artefacts?***

***Reply: This is a good point. Given the high quality of the source databases, artefacts in***

***the form of erroneous data will be few. Like with missing data, we have chosen to defer***

***the dealing with artefacts and potential bias to each specific CDSS project, were the***

***importance and optimal approach to these potential problems can be determined. We***

***now state on P7, second para, that:***

***“Data from the source registers were kept in their exported form with no deletion or***

***curation, and software scripts are used to extract data to form tailor-made new***

***datasets for each specific research project. Data curation or deletion will generally take***

***place in each CDSS project, and only as needed in the original SEM cohort data.”***

***We also state on P11, second para, that:***

***“Bias or errors in the training data will cause a high risk of bias in the final CDSS, but***

***the size and impact of the problem will vary in different CDSS. The optimal approach to***

***the potential problem with bias is therefore best determined in each use case and***

***CDSS. Before clinical implementation, any CDSS based on SEM data should be***

***carefully reviewed and prospectively tested in a clinical trial in the specific healthcare***

***setting.”***

***We think that in such a trial, problems with overfitting are likely to be detected***

***Thank you***

Thanks for reviewing our paper.
